# Growth promotion and disease resistance induced in *Anthurium* colonized by the beneficial root endophyte *Piriformospora indica*

**DOI:** 10.1186/s12870-019-1649-6

**Published:** 2019-01-24

**Authors:** Hui-Feng Lin, Jun Xiong, Hui-Ming Zhou, Chang-Ming Chen, Fa-Zhuang Lin, Xu-Ming Xu, Ralf Oelmüller, Wei-Feng Xu, Kai-Wun Yeh

**Affiliations:** 1 Sanming Academy of Agricultural Sciences, Sanming, Fujian China; 2 College of Life Sciences, Fujian Agriculture and Forestry University, Fuzhou, Fujian China; 3 Institute of Plant Biology, College of Life Science, National Taiwan University, Taipei, Taiwan; 4 Department of General Botany and Plant Physiology, Friedrich-Schiller University, Jena, Germany; 5 Climate Exchange and Sustainable Development Research Center, National Taiwan University, Taipei, Taiwan

**Keywords:** *Anthurium andraeanum*, *Piriformospora indica*, Symbiont, Growth promotion, Disease resistance

## Abstract

**Background:**

*Anthurium andraeanum*, an important ornamental flower, has to go through a growth-delaying period after transfer from tissue culture to soil, which requires time and extra costs. Furthermore, during this period, the plantlets are highly susceptible to bacterial infections, which results in impaired development and severe losses. Here, we aimed to address whether application of the endophytic fungus, *Piriformospora indica* protects the *A. andraeanum* root system during the critical propagation period, and whether *P. indica* reduce the mortality rate by stimulating the host’s resistance against diseases.

**Results:**

We demonstrate that *P. indica* shortens the recovery period of *Anthurium*, promotes growth and confers disease resistance. The beneficial effect of *P. indica* results in faster elongation of *Anthurium* roots early in the interaction. *P. indica*-colonized plants absorb more phosphorus and exhibit higher photosynthesis rates than uncolonized control plants. Moreover, higher activities of stress-related enzymes, of jasmonic acid levels and mRNA levels of jasmonic acid-responsive genes suggest that the fungus prepares the plant to respond more efficiently to potentially upcoming threats, including bacterial wilt.

**Conclusion:**

These results suggest that *P. indica* is a helpful symbiont for promoting *Anthurium* rooting and development. All our evidences are sufficient to support the disease resistance conferred by *P. indica* through the plant-fungal symbiosis. Furthermore, it implicates that *P. indica* has strong potential as bio-fertilizer for utilization in ornamental plant cultivation.

## Background

*Anthurium andraeanum* is a favorite ornamental flower and the second most sold ornamental flower worldwide. Due to its colorful spadix and attractive long-lasting inflorescences, it became a popular cut-flower and pot plant from tropical to temperate areas [1, 2]. To overcome the demerits of conventional propagation, *Anthurium* tissue culture is a powerful tool that complements breeding strategies and accelerates *Anthurium* development [1]. However, transfer of the plantlets to soil is often associated with a high mortality rate [3], since the root system is vulnerable to abiotic and biotic stresses. Therefore, the propagation of *A. andraeanum* by transferring the plantlets from tissue culture to soil is slow, cost intensive and results in severe losses, which hampers the commercial application. *Piriformospora indica* of *Sebacinales* is a well-known endophytic fungus colonizing in the root cortex and epidermal layers of a broad spectrum of plant species, including agronomical, horticultural, medicinal crops and ornamental plants [4]. The mycelium can live and propagate intracellularly and mostly in intercellular space. The endophytic colonization promotes nutrient uptake into the hosts [5, 6], promotes growth [7] and the overall performance of the plants [8, 9]. In this context, it has been suggested that colonization and growth promotion by *P. indica* are independent of plant common symbiotic genes, which rely on different host pathway [8–10]. Moreover, it stimulates the resistance against biotic and abiotic stresses [8, 11]. *P. indica* improves the survival rate of *Chlorophytum* plantlets and promotes the uptake of essential nutrients such as phosphate, copper, iron and zinc [12]. Since the beneficial root colonizer has a high potential for agricultural application based on the working model of *Arabidopsis* and *Brasssica* [13]. We propose whether *P. indica* protects the *A. andraeanum* root system during the critical propagation period, and whether the endophyte can be used to reduce the mortality rate by stimulating the host’s resistance against diseases and other threats during this period. We assayed the growth performance, morphology and fitness of inoculated *A. andraeanum* plantlets and compared these parameters with those of uncolonized controls. RT-PCR was used to determine the expression of plant hormone-stimulated and pathogenesis related genes. We demonstrate that *P. indica* strengthens the plantlets during early phases of propagation in soil. The bigger and stronger plantlets contain higher jasmonic acid (JA) levels and the vulnerable plantlets become more resistant to pathogen attack.

## Methods

### Plant material and fungal culture

*Anthurium andraeanum* cv*.*‘sweet champion’, provided by the Institute of Flower Research (Sanming Academy of Agricultural Sciences, Fujian Province, China) was used in this experiment. Seedlings of about 3 cm height with 2 to 3 adventitious roots were placed in sterilized bottles containing 1/2 MS medium by Murashige and Skoog [14]. After one month, five seedlings per experiment were taken out from the bottle and put plainly on a Petri dish containing 1/2 MS medium. An agar disc (2 cm diameter) of *P. indica* mycelium or an agar disc without fungus (control) were placed at a 1 cm distance from *Anthurium* roots as described by Lee et al. [15]. After 14 days, the inoculated seedlings were transferred to pots with soil in the greenhouse. Further cultivation followed standardized routine conditions. The culture of *Piriformospora indica* (strain DSM11827) used in this study was obtained from the Deutsche Sammlung für Mikroorganismen und Zellkulturen, Braunschweig, Germany (registration number DSM11827). The fungus was firstly identified as a root endophytic fungus by Varma et al. [3, 9]. The culture for working stock was maintained in 4 °C condition, and refreshed by subculturing to new medium every two months. *P. indica* mycelium (20~30 g/l) was inoculated to the roots of the seedlings. The fungal culture was maintained on fresh solid agar medium, and the mycelium used for inoculation was obtained by filtration after cultivation of the fungus in liquid kaefer medium [16].

### Microscopic observation of roots and molecular detection

To confirm whether *P. indica* had performed a successful symbiosis with *Anthurium*, the roots were analyzed by microscopic observation and by RT-PCR analysis. Seven, 14, 21 days after symbiosis with *P. indica*, *Anthurium* roots were either fixed or directly stained with lactophenol/cotton blue in ethanol. The images were taken with an OLYMPUS DP72 (Japan) microscope. For molecular detection, total RNA was isolated from roots of *P. indica*-exposed and control roots using RNeasy plant Mini kit (Qiagen). After reverse transcription, cDNA was synthesized from 1 μg total RNA using Omniscript RT (Qiagen) and Oligo (dT) 20 in 20 μl reaction volume. Primers were designed using the PRIMER BLAST tool from NCBI (http://www.ncbi.nlm.nih.gov/tools/primer- blast) to amplify fragments of the *P. indica Tef* gene for the elongation factor 1a (accession number AJ249911.2; Pi-forward: 5’-TCCGTCGCGCACCATT-3′ and Pi-reverse: 5’-AAATCGCCCTCTTTCCACAA-3′, 84 bp) (Bütehorn et al. 2000). The reaction process was 94 °C 3 min, 94 °C 30 s, 58 °C 30 s, 72 °C 90s, for 30 cycles, and 72 °C for 7 min. Further, PCR products were detected by 1% agarose gel electrophoresis.

### Determination of biomass parameters

The plant height, root length, as well as root, stem and leaf fresh weights of colonized and uncolonized *Anthurium* plants were determined 30, 45 and 60 days after inoculation. The leaf area of *Anthurium* was measured with a leaf area measurement instrument (YMJ-B, Zhejiang TOP, China), and the chlorophyll content by SPAD-502Plus (Konica Minolta Sensing, INC. Japan). Ten samples were analyzed per experiment. The morphological changes of the plant in response to *Ralstonia solanacearum* and *P. indica* colonization were carefully recorded during the growing period. The colonization rate was estimated by microscopic visualization of stained mycelium network covering the area of the root.

### Determination of root growth

The root vigor was determined by TTC (2,3,5-triphenyltetrazolium chloride) methods [17]. The roots were washed with distilled water and patted dry on the filter paper. Root material (0.5 g) was fully immersed in 10 ml solution containing 0.4% TTC and 75 mM phosphate buffer (pH 7.0) and dark heat insulation at 37 °C to 1-3 h before the addition of 2 ml of 1 M sulfate buffer to stop the reaction. The roots were removed, surface-dried and homogenized in 3–4 ml ethyl acetate before grinding with mortar and pestle. The red extract solution was transferred to a test tube, and the residue washed 2~3 times with a small amount of ethyl acetate. The combined ethyl acetate phases were added up to 10 ml for spectrophotometric measurement at 485 nm. Calibration was performed with a standard curve to quantify the amount of tetrazolium, which was reduced in the reaction. For each growth stage, three replicates were performed.

### Determination of nutrient content in plant organs

Previously published protocols were used to determine the nitrogen, phosphorous and potassium contents of *Anthurium* root, stems, and leaves [18]. For assessment of nutrient content, a total 10 independent *P. indica*-colonized and -uncolonized *Anthurium* seedlings were used to determine various plant parts on 30, 45 and 60 days, respectively.

### Activity assay of antioxidant enzymes

Leaf material without the midrib (0.1 g) was homogenized in 2 ml 50 mM PBS buffer (137 mM NaCl, 2.7 mM KCl, 10 mM sodium phosphate dibasic, 2 mM potassium phosphate monobasic, pH 7.8) containing 0.2 mM ethylenediamine tetraacetic acid (EDTA) and 1% polyvinylpyrrolidone (PVP). The homogenate was centrifuged at 24,000- × *g* for 20 min and the supernatant was used for the enzyme assays. Superoxide dismutase (SOD) activity was assayed [19] by measuring the reduction of nitrotetrazolium blue chloride at 560 nm. The catalase (CAT) activity was determined spectrometrically as described [20] by measuring H_2_O_2_ consumption at 240 nm, and the peroxidase (POD) activity was determined by absorption changes at 470 nm as previously described [21].

### Analysis of JA levels in leaves

Ten independent *Anthurium* seedlings were taken. 2 g of leaves were weighed, frozen immediately in liquid nitrogen and were then grinded in 100 mM PBS buffer pH 7.4 at 2 °C before centrifugation at 3000 rpm for 20-min. The JA content was detected with the Plant Jasmonic Acid (JA) ELISA Kit (My BioSource, San Diego, CA, USA). For the determination of the effects of pathogens on the JA content in *Anthurium* leaves, the JA content was assayed 12, 24, and 48 h after inoculation of the plants with the bacterium *R. solanacearum* 004. Plants were surface-sterilized with 70% alcohol and air-dried. The bacterial suspension of *R. solanacearum* 004 (100 CFU/ml) was injected into the midvein of the latest mature leaf. Sterile demineralized water was used as a negative control.

### Analysis of gene expression by RT-qPCR

Total RNA from root tissues was extracted with a protocol previously described for pine tree seedlings [15]. The complementary DNA (cDNA) syntheses, the RT-qPCR conditions and gene-specific primers are given in Table 1. Expression values were normalized using the housekeeping gene *GAPDH*.

**Table 1:** Primer sequences used for this study

Gene name	Related pathway	Sequence (5′-3′)
*NPR1(Nonexpressor of PR genes 1–3)*	SA	F:5’-GACAAAGTTGTTATAGAGGACA-3′
		R:5’-CTTTAACAAGCTCTTCCGGC-3’
*VSP (vegetative storage protein)*	JA	F:5’-CATAGACTTCGACACGGTGC-3’
		R:5’-AGGAGGGTATCATCTAGGTCA-3’
*ERF (Ethylene responsive factor)*	ET	F:5’-GCAGCCCTCGTTGTAGAGAG-3’
		R:5’-GAAGGTGGACTGGAGGGAGA-3’
*LOX2 (Lipoxygenase 2)*	JA	F:5’-TTGACCCAACCAAGCGGATA-3’
		R:5’-TTACCGGACGAGCAAGTTCA-3’
*PR5 (Pathogenesis-related protein 5)*	SA	F:5’-CCCGTCACTCTGGCTGAAT-3’
		R:5’-TGACCTTAAGCATGTCGGGG-3’
*PR1(Pathogenesis-related protein 1)*	SA	F:5’-CGAAAGCTCAAGATAGCCCAC-3’
		R:5’-CCAGGCTAAGTTTTCCCCGT-3’
*GAPDH*	Housekeeping gene	F:5’-GTCGATGTATCAGTCGTTGATCTTACT-3’
		R:5’-CGAGCTTAACAAAAGAGTCGTTCAAG-3’

F: Forward primer; R: Reverse primer

### Statistical analyses

Data display means with standard errors of three independent biological samples. Two-way analysis of variance (ANOVA) was used to evaluate the differences in gene expression between colonized and non-colonized roots*.* GraphPad Prism version 5.01 software (2007) was used for statistical analysis. In all graphs, the error bars indicate the standard deviation.

## Results

### Colonization of plant roots by *P. indica*

**Fig 1:**
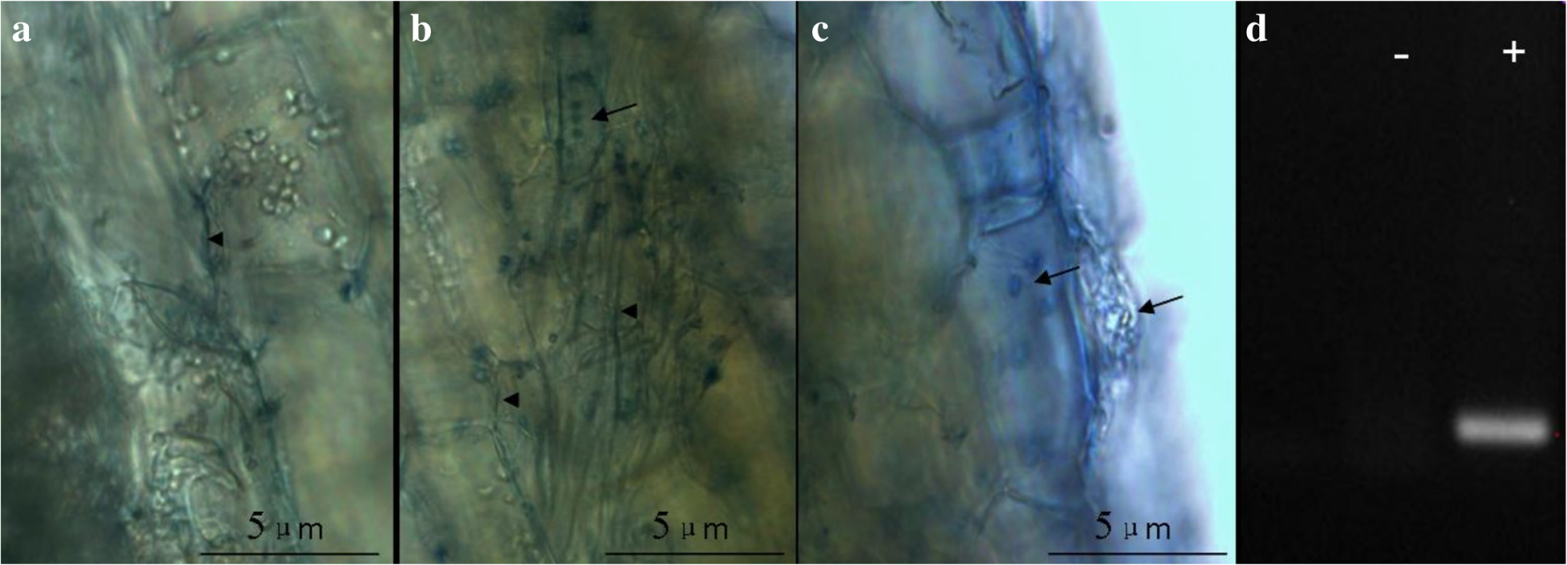
Stainings of *P. indica* hyphae and spores in the roots of *Anthurium* seedlings inoculated for 2 weeks. **a**, **b** and **c**: Microscopic observation of hyphae and spores in *P. indica*-colonized roots. **d**: showing Pitef mRNA detected in *P. indica* colonized (+) but not in *P. indica* -uncolonized (−) roots. Scale bar indicates 5 μm for each of the three pictures. Arrow indicates chlamydospores; arrowhead indicates hyphae; Pitef: Translational elongation factor genes of *P. indica*

Healthy *Anthurium* seedlings with actively growing adventitious roots were inoculated with *P. indica* following the method previously described [15]. Successful root colonization was checked by microscopic examination. 14 days after inoculation, the roots were extensively colonized by *P. indica* (Fig. 1). The fungal hyphae passed through the root epidermal cellular layers and multiplied in the cortex layers; spores were copiously present in the cortex layer (Fig. 1). PCR analysis with the *Pitef* marker gene further confirmed colonization of the root tissue (Fig. 1). Subsequently, the colonized and control *Anthurium* plants were moved from agar plates to pots in the greenhouse.

### *P. indica* stimulates biomass production of *Anthurium*

**Fig. 2:**
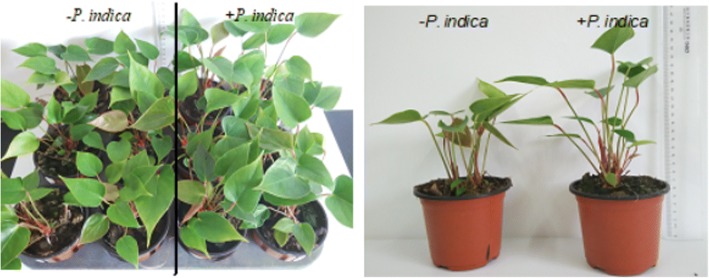
Effect of *P. indica* on the growth of *Anthurium* in pots at 30 days after inoculation. The colonization of *P. indica* led to biomass increase in *Anthurium* plant

The sizes of roots and shoots, as well as of the fresh weights and leaf areas of *P. indica*-colonized *Anthurium* plants were investigated 30, 45 and 60 days after the transfer to pots. As shown in the Fig. 2, *P. indica* promoted growth of the seedlings, the number of branches, the heights and surface areas of the leaves and the lengths of the roots were increased compared to uncolonized seedlings (Table 2). Also the fresh weights of roots, stems and leaves increased in the presence of the fungus. The beneficial effect of the fungus is particularly striking during the early phase (30 days), i.e. during the critical recovery phase after transfer of the seedlings to pots, while, the parameters for uncolonized seedlings catch up during later growth phases, although a growth promoting effect of the fungus is still detectable. The chlorophyll and total protein contents of *P. indica*-colonized *Anthurium* leaves were also higher than those of the uncolonized controls (Tables 2, 3). Compared to leaves, the increases in fresh weights were stronger for the roots. In summary, the stimulatory effect of *P. indica* is detectable during the critical early phase after transfer of the seedlings to pots.

**Table 2:** Growth parameters of *Anthurium* after *P. indica* colonization from tissue culture to greenhouse

Days after transfer	Treatment	Root length (cm)	Plant height (cm)	Root FW (g)	Stem FW (g)	Leaf FW (g)	Chlorophyll (mg/g)	Area of 2nd leaf (cm^2^)	Area of 3rd leaf (cm^2^)	Root vigor (mg/g·h^−1^)
30	*- P. indica*	5.0 ± 0.3^b^	10.5 ± 0.2^b^	0.4 ± 0.1^b^	1.2 ± 0.1^b^	0.7 ± 0.0^b^	47.4 ± 0.5^b^	8.7 ± 0.6^b^	8.5 ± 0.6^b^	124.4 ± 0.7^b^
	*+ P. indica*	8.6 ± 0.2^a^	12.8 ± 0.3^a^	1.2 ± 0.2^a^	2.1 ± 0.2^a^	1.5 ± 0.2^a^	51.5 ± 0.2^a^	15.6 ± 1.1^a^	14.7 ± 1.1^a^	527.4 ± 1.6^a^
45	*- P. indica*	5.8 ± 0.8^b^	12.1 ± 0.5^b^	1.5 ± 0.3^a^	1.7 ± 0.3^a^	1.1 ± 0.2^b^	45.7 ± 0.5^b^	8.2 ± 0.8^b^	10.2 ± 1.4^b^	145.8 ± 2.0^b^
	*+ P. indica*	11.7 ± 0.9^a^	14.1 ± 0.4^b^	1.7 ± 0.2^a^	2.6 ± 0.2^a^	1.7 ± 0.2^a^	48.4 ± 0.5^a^	14.4 ± 1.2^a^	13.7 ± 1.6^a^	594.0 ± 1.8^a^
60	*- P. indica*	10.9 ± 0.7^b^	13.0 ± 0.2^b^	3.2 ± 0.4^a^	2.3 ± 0.2^b^	1.6 ± 0.1^b^	43.0 ± 0.6^b^	12.3 ± 0.9^b^	10.4 ± 0.8^a^	152.1 ± 1.3^b^
	*+ P. indica*	14.2 ± 0.7^a^	17.0 ± 0.5^a^	3.5 ± 0.5^a^	3.3 ± 0.3^a^	2.1 ± 0.2^a^	48.1 ± 0.6^a^	16.2 ± 0.9^a^	16.1 ± 1.2^a^	612.1 ± 3.9^a^

Data are based on 3 independent experiments. The errors are SDs. Values in a column followed by different letters are significantly different at *P* < 0.05 according to the Duncan’s multiple range test

Significant differences (*p*<0.05) are indicated in different letters (a, b)

**Table 3:** Estimation of soluble protein and antioxidative enzymes of *Anthurium* after *P. indica* colonization from tissue culture to greenhouse

Days after transfer	Treatment	Soluble protein (μg ^−1^ FW)	SOD (U g^−1^ FW)	POD (U g^−1^ FW)	CAT(U·g^−1^ FW)
30	*- P. indica*	25.1 ± 0.4^b^	87.7 ± 2.6^b^	160.7 ± 9.5^b^	106.3 ± 0.3^b^
	*+ P. indica*	37.1 ± 0.3^a^	101.7 ± 2.4^a^	198.7 ± 3.1^a^	136.5 ± 1.6^a^
45	*- P. indica*	28.5 ± 0.4^b^	98.7 ± 2.5^b^	172.6 ± 16.0^b^	113.5 ± 0.4^b^
	*+ P. indica*	41.7 ± 0.3^a^	115.3 ± 6.9^a^	208.7 ± 7.7^a^	140.2 ± 0.6^a^
60	*- P. indica*	30.5 ± 0.3^b^	102.0 ± 1.4^b^	174.2 ± 9.2^b^	116.8 ± 2.2^b^
	*+ P. indica*	43.5 ± 1.8^a^	116.9 ± 1.5^a^	211.7 ± 11.0^a^	145.5 ± 1.8^a^

Data are based on 3 independent experiments. The errors are SDs. Values in a column followed by different letters are significantly different at *P* < 0.05 according to the Duncan’s multiple range test

Significant differences (*p*<0.05) are indicated in different letters (a, b)

### *P. indica* stimulated P, but not N and K uptake

To determine whether the fungal effect on growth is reflected by an increase in nutrient uptake, we determined the P and N content in the roots, stems and leaves of colonized and non-colonized plants and compared the results with the K uptake (Table 4). Interestingly, neither N, nor K uptake was promoted by the fungus in roots, stems and leaves at the three investigated time points, while the P content was higher in all three organs at the three time points in the presence of the fungus (Table 4). The strongest effect was observed from leaves. Consistent with the results for the biomasses, we also observed that the stimulatory effect of the fungus on the P content had its maximum 30 days after transfer of the seedlings to pots. Thus, it appears that *P. indica* specifically promotes P uptake.

**Table 4:** Assessment of *P. indica* effect on nutrient contents of *Anthurium* from the tissue culture to greenhouse

		N (μg g^−1^ FW)		P (μg g^−1^ FW)		K (μg g^−1^ FW)	
		-*P*.indica	+*P*. indica	-*P*.indica	+*P*. indica	-*P*.indica	+*P*. indica
30d	root	3.83 ± 0.33^a^	3.49 ± 0.18^a^	1.25 ± 0.04^b^	1.16 ± 0.07^a^	0.10 ± 0.00^a^	0.12 ± 0.01^a^
	stem	3.07 ± 0.06^a^	3.21 ± 0.19^a^	0.48 ± 0.02^b^	0.66 ± 0.02^a^	0.09 ± 0.00^a^	0.12 ± 0.01^a^
	leaf	3.08 ± 0.06^a^	3.07 ± 0.07^a^	0.24 ± 0.01^b^	0.40 ± 0.02^a^	0.01 ± 0.00^a^	0.01 ± 0.00^a^
45d	root	3.33 ± 0.16^a^	3.33 ± 0.07^a^	1.28 ± 0.02^b^	1.41 ± 0.01^a^	0.08 ± 0.00^a^	0.10 ± 0.00^a^
	stem	3.31 ± 0.08^a^	3.34 ± 0.08^a^	0.76 ± 0.02^b^	0.82 ± 0.01^a^	0.09 ± 0.00^a^	0.10 ± 0.00^a^
	leaf	3.28 ± 0.10^a^	3.49 ± 0.05^a^	0.39 ± 0.01^b^	0.49 ± 0.01^a^	0.07 ± 0.00^a^	0.08 ± 0.00^a^
60d	root	3.23 ± 0.12^a^	3.38 ± 0.53^a^	1.33 ± 0.04^b^	1.47 ± 0.02^a^	0.10 ± 0.00^a^	0.11 ± 0.00^a^
	stem	3.38 ± 0.07^a^	3.35 ± 0.17^a^	0.83 ± 0.01^b^	0.91 ± 0.03^a^	0.10 ± 0.00^a^	0.12 ± 0.00^a^
	leaf	3.56 ± 0.19^a^	3.36 ± 0.18^a^	0.47 ± 0.02^a^	0.57 ± 0.02^a^	0.07 ± 0.00^a^	0.10 ± 0.00^a^

Data are based on 3 independent experiments. The errors are SDs. Values in a column followed by different letters are significantly different at *P* < 0.05 according to the Duncan’s multiple range test

Significant differences (*p*<0.05) are indicated in different letters (a, b)

### *P. indica* promotes antioxidative enzyme activities and the expression of defense-associated genes

**Fig. 3:**
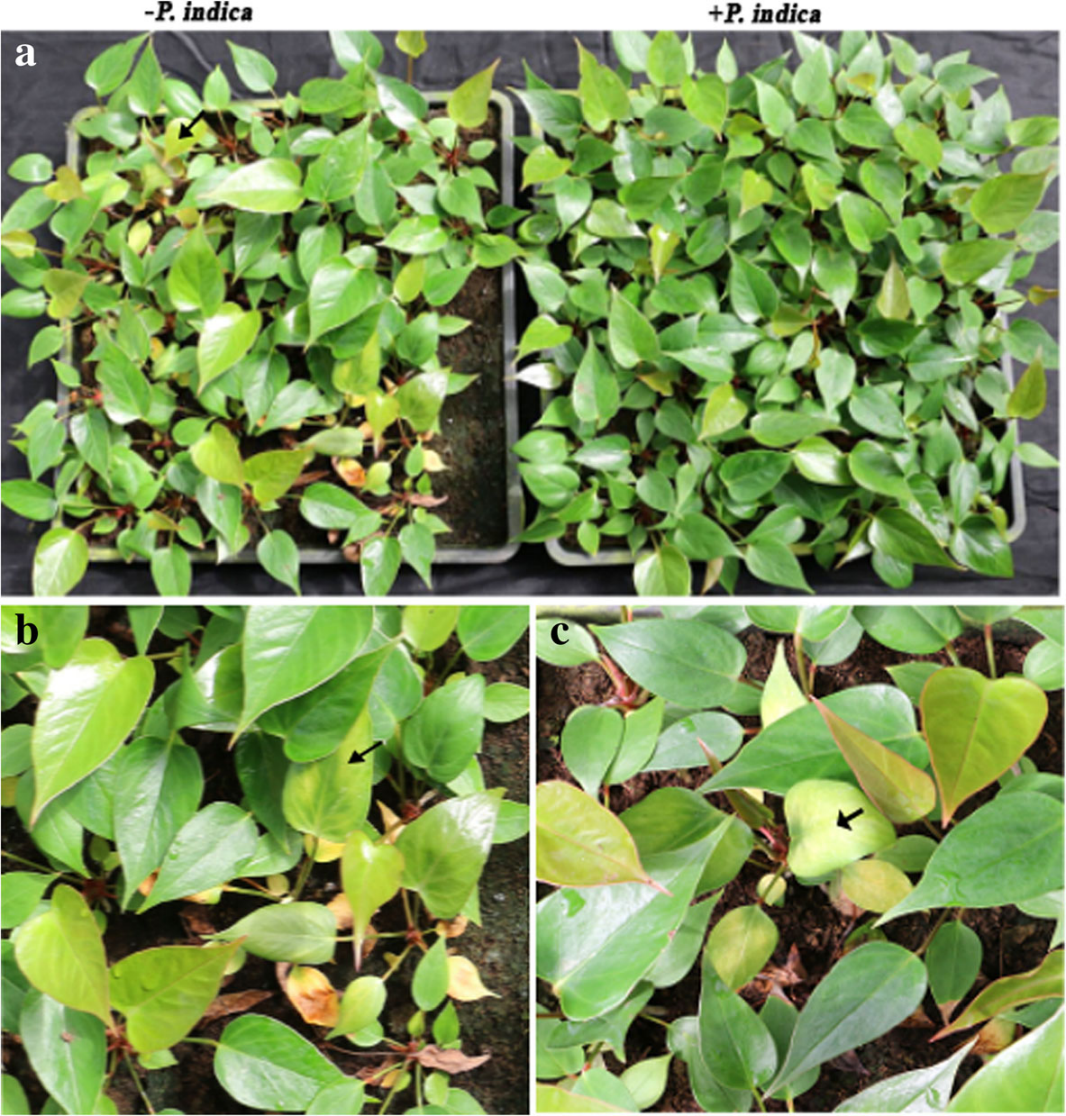
Performance of uncolonized (-) and colonized (+) *Anthurium* plants at 60 days after inoculation. **a** picture shows *P. indica* uninoculated (-) (left) and *P. indica* inoculated (+) (right) plants grown in greenhouse. **b** and **c** indicate *P. indica* -unincoulated plants. Arrow shows the incidence of early wilt disease symptom after moving *Anthurium* from tissue culture to pots in greenhouse

Figure 3 shows that *Anthurium* plants are quite susceptible to bacterial infection during the first period on soil in the greenhouse. This is a severe problem for the breeders since these infections are responsible for the high plant loss. *P. indica-*colonized *Anthurium* plants are visibly more resistant to biotic stresses (Fig. 3). Better performance of the colonized plants is associated with higher activities of antioxidative enzymes. Overall, we observed an approximately 20% increase in the activities of SOD, CAT and POD of the colonized plants at the three time points (Table 3).

### *P. indica* stimulates JA accumulation and hormone-response genes upon *R. solanacearum* infection

**Fig. 4:**
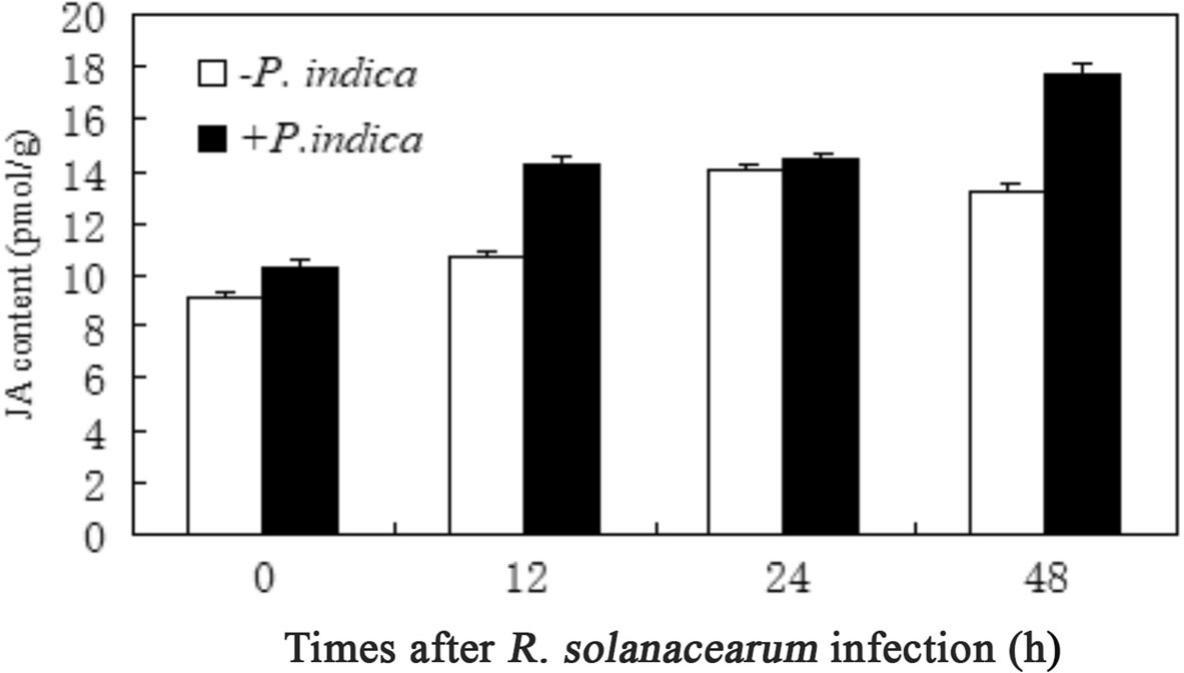
The JA level in leaves of *Anthurium* colonized with or without *P. indica*. JA level was assayed on 0, 12, 24 and 48 h after *R. solanacearum* infection. Bar represents the mean ± standard error of the mean

**Fig. 5:**
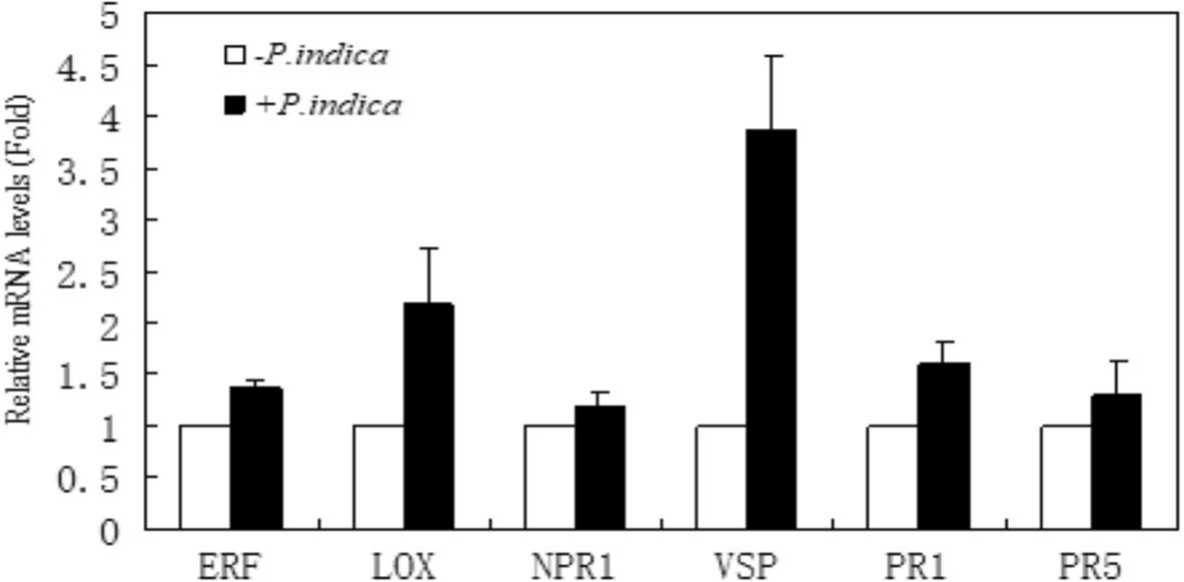
Expression of hormone-responsive genes in leaves of *P. indica* -uncolonized (−) and colonized (_+_) *Anthurium* plants at 2 days after challenging with *R. solanacearum*. The relative mRNA levels of ET-responsive *ERF*, JA-responsive *VSP*, *LOX*, and SA-responsive *PR1*, *PR5* and *NPR1*, were analyzed by quantitative RT-PCR. Expression levels were calculated relative to the constitutive *GAPDH* mRNA level. Bar represents the mean ± standard error of the mean

Before infection, the JA content in the leaves was already slightly higher compared to plants, which were not exposed to the fungus (Fig. 4). This may support the activation of the defense response during symbiotic interaction with *P. indica*. Moreover, the JA level was enhanced in parallel with bacterial infection. Upon infection of the roots at 12 h, 1, 2 days with *R. solanacearum*, the JA-responsive genes, such as *VSP (*Vegetative storage protein)*, NPR 1* (Non-expresser of Pathogenesis-Related) and *LOX* (Lipoxygenase), increased and this increase was stronger for the colonized plants as compared to the uncolonized controls (Fig. 5). Both PR1 (Pathogenesis-Related 1) and PR5 (Pathogenesis-Related 5) were also increased in the *P. indica*-colonized plants (Fig. 5).

## Discussion

*P. indica* colonizes a broad spectrum of plant species including agriculturally important monocots to dicots [22, 23]. The present study established a new symbiotic system between *Anthurium* and *P. indica*. Comparable to observations with *Arabidopsis thaliana* [24], *Brassica napus* [25], *Oryza sativa* [26], *Triticum aestivum* [27] or *Medicago truncatula* [28], the fungus stimulates biomass accumulation and in particular root growth and length of *Anthurium*. Since the roots of *Anthurium* are partially growing on the soil surface, the effects of the endophyte on these parts of the roots are difficult to quantify, however if the entire root system is considered a clear growth promotion is detectable. The effects of *P. indica* on the morphology of the *Anthurium* roots correlated with the observed growth stimulation of the fast root elongation as well as the increasing size of leaf and stem, which occur preferentially during the first 30 days after transfer to soil. During later stages, the beneficial effects decline, which was also observed for barley [29, 30], described this phenomenon as “initial advantages of the host plant”. This is particularly important for *Anthurium* seedlings, since they are extremely sensitive to stress and diseases after transfer from tissue culture to pots. Introduction of *P. indica* into the commercially applied propagation process could therefore be a helpful tool to overcome the problems of the breeders. While this investigation focusses on the early period of *Anthurium* growth, it is reasonable to assume that *P. indica* also supports later steps in the development of the ornamental plant. Earlier flowering of colonized *A. thaliana* plants suggests that the basic metabolism of the plant is stimulated by the fungus throughout the plant’s life [31].

Hyphae take up phosphate (P) from the soil via the fungal PTP transporter and transfer the nutrient to the host roots, as shown for maize [6]. The fungus also improves the activity of acid and alkaline phosphatases in soil [26, 32] which allows better access to P in the soil. Considering the effects of *P. indica* on nutrient, in particular P uptake into the hosts and the growth responses published for various plant species, it is difficult to predict a putative correlation. The endophyte did not promote P uptake into *Cyclamen* under low P conditions, but increased the P content of the plant under normal P supply [33]. On the other hand, the endophyte increased the biomass and yield in tobacco but did not promote P accumulation [34]. Similar results were described for mung bean [35] and barley [29]. Therefore, the growth promoting effects of *P. indica* on some host plants did not correlate with P uptake rates. Our study showed that *P. indica* promotes P accumulation in *Anthurium*, and the accumulation of P in leaves is higher than in the roots and stems. Interestingly, the N and K contents in the plant were not stimulated by the fungus, demonstrating specificity for P. The plant P supply improves the sensitivity of CO_2_ assimilation and the N content, which in turn improves the efficiency of photosynthesis and the growth rate [36]. However, in *Anthurium*, the fungus increased the chlorophyll level, but not the N uptake rate. Also Achatz et al. [29] showed that *P. indica* increased photosynthetic efficiency under the low light intensity conditions while N accumulation was not affected. *P. indica* also increased the photosynthetic efficiency in maize [37], but no nutrient uptake data are available under the same conditions. Higher chlorophyll levels and thus photosynthesis rates might accelerate carbohydrate synthesis and thus growth of *A. andraeanum*.

Numerous studies also suggest a relationship between *P. indica* and the host systemic antioxidant capacity has been reviewed by Franken [38], but again no clear picture emerges from the literature data. In barley, the ascorbic acid level in roots increased while the amount of ascorbic acid in leaves was not affected by *P. indica* [8]. Vadassery et al. [39] also showed elevated ascorbic acid and glutathione levels in *Arabidopsis* colonized by *P. indica.* In our study, the SOD, CAT and POD activities which are central for plant cell antioxidation [40] were stimulated by the fungus. The better performance of the plants with effective antioxidation activity suggests that the fungus strengthens the innate immunity and abiotic stress response during the critical growth period after transfer of the plantlets from tissue culture to pots. Baltruschat et al. [30] showed an increase in 5 antioxidant enzymes in colonized barley roots under salt stress. Protective enzymes are not only involved in the defense response of plants, but also play an important role in their general metabolism [41]. This might explain why colonized *A. andraeanum* plants perform better. Since the stronger plantlets (cf. also [8, 42, 43] showed reduced morbidity rate (Fig. 3), *P. indica* might be a power tool for future applications in the propagation of *Anthurium*.

Disease resistance pathways had been well revealed in rice, barley and *A. thaliana* [8, 31, 44]. JA and SA signaling pathway in barley leaves have no response to powdery mildew resistant in colonized barley, heat shock protein HSP70 gene and pathogenesis related genes HvPr17 were responsive for the powdery mildew resistance in barley [45]. However, in *A. thaliana,* the systemic resistance response was independent of salicylate signaling, but required an operative jasmonate defense pathway [46]. Vahabi et al. [47] also confirmed that the exudates of hyphal induced high JA/ABA/SA/JA-Ile levels in roots and stems of *A. thaliana* by means of transcriptome and HPLC-MS techniques. It was reported that *P. indica* is capable of inducing resistance in *Arabidopsis* in response to infection of leaf pathogens of *Gonovilomyces orontii*. In this study, to test whether *P. indica*-colonized *Anthurium* plants are better prepared for infections, we used the bacterial *R. solanacearum*, which frequently causes severe plant losses by root rot during the propagation procedure in agriculture. The pathogenic bacterium causes wilts, which are associated with a JA-dependent defense response of the hosts. These reports suggested that the mechanisms of different plants respond to colonization are different. In our study, the relative level of PR gene in colonized *Anthurium* leaves were increased, suggesting that SA/ETH (Ethylene)/VSP involved in plant hormone response are in leaves of *Anthurium*, which are sensitive to bacterial diseases infection.

Pathogenesis-related (PR) proteins (SA-responsive related genes) play an important role in the disease resistance response [48]. Our study showed that *P. indica* promote both the PR1 and PR5 significantly up-regulated in *Anthurium* leaves about 0.5-fold levels. This suggests that the endophyte plays the induced systemic resistance mechanism (ISR) in plant to activate JA-dependent defense processes upon bacterial infection [46]. Because of coordination or antagonism of different signaling pathways, PR genes can be regulated by several plant hormone signaling molecules when plants encounter the biotic and abiotic stresses [49, 50]. JA/SA and other signaling molecules levels related to systemic acquired resistance (SAR) inducing the relative mRNA levels of PR genes had been investigated. Rout et al. [51] showed that the PR5 levels in garlic were enhanced when plant was suffered exogenous hormones SA/ABA/MeJA (methyl jasmonate)/ETH and drought, salt damage and wound conditions that explained the external hormone accumulation related to plant acquired resistance. Li et al. [44] revealed that the PR5 levels in leaves increased after spraying ABA at the root of rice. VSP is a subset of JA-regulated defense gene, is more strongly expressed against the pathogen challenge. Enhanced VSP expression was detected in *P. indica*-mediated resistance to powdery Mildew in *A. thaliana* [46].

Different from the levels of above genes up-regulated after *P. indica* infected, the JA related gene lipoxygenase (LOX) did not change significantly whereas JA-inducible vegetative storage protein (VSP) levels were significantly enhanced. JA is an identifying signal molecule in plant perception of stress, and responds to various plant stress responses through long distance transmission through sensory vascular bundles [52]. Further, the systemic resistance response was independent of salicylate signaling, but required an operative jasmonate defense pathway [46]. Moreover, the biosynthesis efficiency of JA was affected by various factors: over expression of JA synthesis key enzyme gene AOX (alternative oxidase) in transgenic *Arabidopsis* and tomato plants did not increase in contrast to wild type, which is associated with the substrate concentration. Similarly, the expression of JA in the injured plant were also not obvious related to the expression of the JA-related key enzyme OPDA, the biosynthesis of JA in *Arabidopsis* and tomato plants was restricted by substrate concentration or other factors other than OPDA [53]. Therefore, *P. indica* did not induce the expression levels of genes related to JA synthesis in barley [8], nor can’t reflect the JA content in plants. In our study, the JA content of *P. indica*-colonized plants increased significantly, but the expression of LOX did not significantly increase, which also shows that JA synthesis is affected by other factors besides LOX gene after the infection of bacterial diseases in *Anthurium*. The JA content in *P. indica*-colonized *Anthurium* leaves showed a single peak variation within 48 h, suggesting that JA is an early molecular signal for the interaction between *Anthurium* and its pathogenic bacteria. Vahabi et al. [47] showed that the regulation of JA was restricted when the plants and microorganisms were mutual discerned. The JA content in leaves was continued increasing, enhanced the root–rot disease resistance, which indicates that *P. indica* can be used as probiotics to improve the resistance of *Anthurium*. In addition, in our study, the levels of *ERF* (Ethylene responsive factor) gene that was an ethylene responsive transcription factor were enhanced, and JA could act on ethylene signaling by regulating ERF1 [54]. This was consistent with the results that the content of JA in the root, stem and leaf of *P. indica-*colonized *Anthurium* tissues was higher than that of control plants, respectively.

## Conclusion

In summary, our results indicated that *P. indica* could evoked multiple signaling pathways involved in the defense function of *A. andraeanum*. However, the interacting mechanism between signaling pathways, regulation of gene expression, and the effects of hormone levels is still necessary for further investigation. Moreover, our result implicates that it is promising to develop *P. indica* as a biofertilizer for application in ornamental plants.

## Abbreviations

ABA: Abscisic acid
AOX: Alternative oxidase
AOX: Alternative oxidase
CAT: Catalase
ETH: Ethylene
ISR: Induced systemic resistance
JA: Jasmonic acid
LOX: Lipoxygenase
NPR1: Non-expresser of PR
OPDA: Oxo-Phytodienoic Acid
POD: peroxidase
PR: Pathogenesis-related
RT-PCR: Reverse transcription polymerase chain reaction
SA: Salicylic acid
SOD: Superoxide dismutase
VSP: Vegetative storage protein

## References

[1] Teixeira da Silva JA, Dobránszki J, Winarto B, Zeng S (2015). *Anthurium* in vitro: a review. Sci Hortic.

[2] Elibox W, Umaharan P (2010). Cultivar differences in the deterioration of vase-life in cut-flowers of *Anthurium andraeanum* is determined by mechanisms that regulate water uptake. Sci Hortic.

[3] Varma A, Savita V, Sudha, Sahay N, Butehorn B, Franken P (1999). *Piriformospora indica*, a cultivable plant-growth-promoting root endophyte. Appl Environ Microbiol.

[4] Ye W, Shen CH, Lin Y, Chen PJ, Xu X, Oelmuller R, Yeh KW, Lai Z (2014). Growth promotion-related miRNAs in Oncidium orchid roots colonized by the endophytic fungus *Piriformospora indica*. PLoS One.

[5] Sherameti I, Shahollari B, Venus Y, Altschmied L, Varma A, Oelmuller R (2005). The endophytic fungus *Piriformospora indica* stimulates the expression of nitrate reductase and the starch-degrading enzyme glucan-water dikinase in tobacco and Arabidopsis roots through a homeodomain transcription factor that binds to a conserved motif in their promoters. J Biol Chem.

[6] Yadav V, Kumar M, Deep DK, Kumar H, Sharma R, Tripathi T, Tuteja N, Saxena AK, Johri AK (2010). A phosphate transporter from the root endophytic fungus *Piriformospora indica* plays a role in phosphate transport to the host plant. J Biol Chem.

[7] Bagde US, Prasad R, Varma A (2011). Influence of culture filtrate of *Piriformospora indica* on growth and yield of seed oil in Helianthus annus. Symbiosis.

[8] Waller F, Achatz B, Baltruschat H, Fodor J, Becker K, Fischer M, Heier T, Huckelhoven R, Neumann C, von Wettstein D (2005). The endophytic fungus *Piriformospora indica* reprograms barley to salt-stress tolerance, disease resistance, and higher yield. Proc Natl Acad Sci U S A.

[9] Varma A, Sowjanya Sree K, Arora M, Bajaj R, Prasad RC, Kharkwal A (2014). Funnctions of novel symbiotic fungus - *Piriformospora Indica*, vol. 80.

[10] Banhara A, Ding Y, Kühner R, Zuccaro A, Parniske M (2015). Colonization of root cells and plant growth promotion by *Piriformospora indica* occurs independently of plant common symbiosis genes. Front Plant Sci.

[11] Harrach BD, Baltruschat H, Barna B, Fodor J, Kogel KH (2013). The mutualistic fungus *Piriformospora indica* protects barley roots from a loss of antioxidant capacity caused by the necrotrophic pathogen Fusarium culmorum. Mol Plant-Microbe Interact.

[12] Gill SS, Gill R, Trivedi DK, Anjum NA, Sharma KK, Ansari MW, Ansari AA, Johri AK, Prasad R, Pereira E (2016). *Piriformospora indica*: potential and significance in plant stress tolerance. Front Microbiol.

[13] Varma A, Sherameti I, Tripathi S, Prasad R, Das A, Sharma M, Bakshi M, Johnson JM, Bhardwaj S, Arora M, Hock B (2012). 13 the symbiotic fungus Piriformospora indica: review. Fungal Associations.

[14] Murashige T, Skoog F (2006). A revised medium for rapid growth and bio assays with tobacco tissue cultures. Physiol Plant.

[15] Lee YC, Johnson JM, Chien CT, Sun C, Cai D, Lou B, Oelmuller R, Yeh KW (2011). Growth promotion of Chinese cabbage and *Arabidopsis* by *Piriformospora indica* is not stimulated by mycelium-synthesized auxin. Mol Plant-Microbe Interact.

[16] Hill T, Käfer E (2001). Improved protocols for Aspergillus minimal medium: Trace element and minimal medium salt stock solutions, vol. 48.

[17] Lopez Del Egido L, Navarro-Miro D, Martinez-Heredia V, Toorop PE, Iannetta PPM (2017). A spectrophotometric assay for robust viability testing of seed batches using 2,3,5-Triphenyl Tetrazolium chloride: using Hordeum vulgare L. as a model. Front Plant Sci.

[18] Lowther JR (1980). Use of a single sulphuric acid - hydrogen peroxide digest for the analysis of Pinus radiata needles. Commun Soil Sci Plant Anal.

[19] Giannopolitis CN, Ries SK (1977). Superoxide Dismutases: II. Purification and quantitative relationship with water-soluble protein in seedlings. Plant Physiol.

[20] Gao Y, Sun X, Sun Z, Zhao N, Li Y (2008). Toxic effects of enrofloxacin on growth rate and catalase activity in Eisenia fetida. Environ Toxicol Pharmacol.

[21] Cakmak I, Marschner H (1992). Magnesium deficiency and high light intensity enhance activities of superoxide dismutase, ascorbate peroxidase, and glutathione reductase in bean leaves. Plant Physiol.

[22] Sun C, Johnson JM, Cai D, Sherameti I, Oelmuller R, Lou B (2010). *Piriformospora indica* confers drought tolerance in Chinese cabbage leaves by stimulating antioxidant enzymes, the expression of drought-related genes and the plastid-localized CAS protein. J Plant Physiol.

[23] Qiang X, Weiss M, Kogel KH, Schafer P (2012). *Piriformospora indica*-a mutualistic basidiomycete with an exceptionally large plant host range. Mol Plant Pathol.

[24] Peškan-Berghöfer T, Shahollari B, Giong Pham H, Hehl S, Markert C, Blanke V, Kost G, Varma A, Oelmüller R (2004). Association of *Piriformospora indica* with *Arabidopsis thaliana* roots represents a novel system to study beneficial plant–microbe interactions and involves early plant protein modifications in the endoplasmic reticulum and at the plasma membrane. Physiol Plant.

[25] Su ZZ, Wang T, Shrivastava N, Chen YY, Liu X, Sun C, Yin Y, Gao QK, Lou BG (2017). *Piriformospora indica* promotes growth, seed yield and quality of Brassica napus L. Microbiol Res.

[26] Das J, Ramesh KV, Maithri U, Mutangana D, Suresh CK (2014). Response of aerobic rice to *Piriformospora indica*. Indian J Exp Biol.

[27] Hosseini F, Mosaddeghi MR, Dexter AR (2017). Effect of the fungus *Piriformospora indica* on physiological characteristics and root morphology of wheat under combined drought and mechanical stresses. Plant Physiol Biochem.

[28] Li L, Li L, Wang X, Zhu P, Wu H, Qi S (2017). Plant growth-promoting endophyte *Piriformospora indica* alleviates salinity stress in Medicago truncatula. Plant Physiol Biochem.

[29] Achatz B, Kogel KH, Franken P, Waller F (2010). *Piriformospora indica* mycorrhization increases grain yield by accelerating early development of barley plants. Plant Signal Behav.

[30] Baltruschat H, Fodor J, Harrach BD, Niemczyk E, Barna B, Gullner G, Janeczko A, Kogel KH, Schafer P, Schwarczinger I (2008). Salt tolerance of barley induced by the root endophyte *Piriformospora indica* is associated with a strong increase in antioxidants. The New phytologist.

[31] Kim D, Abdelaziz ME, Ntui VO, Guo X, Al-Babili S (2017). Colonization by the endophyte Piriformospora indica leads to early flowering in Arabidopsis thaliana likely by triggering gibberellin biosynthesis. Biochem Biophys Res Commun.

[32] Ngwene B, Boukail S, Söllner L, Franken P, Andrade-Linares DR (2016). Phosphate utilization by the fungal root endophyte *Piriformospora indica*. Plant Soil.

[33] Ghanem G, Ewald A, Zerche S, Hennig F (2014). Effect of root colonization with Piriformospora indica and phosphate availability on the growth and reproductive biology of a Cyclamen persicum cultivar. Sci Hortic.

[34] Barazani O, Benderoth M, Groten K, Kuhlemeier C, Baldwin IT (2005). *Piriformospora indica* and *Sebacina vermifera* increase growth performance at the expense of herbivore resistance in Nicotiana attenuata. Oecologia.

[35] Ray JG, Valsalakumar N (2010). Arbuscular mycorrhizal fungi and *Piriformospora indica* individually and in combination with *Rhizobium* on green grain. J Plant Nutr.

[36] Walker Anthony P, Beckerman Andrew P, Gu L, Kattge J, Cernusak Lucas A, Domingues Tomas F, Scales Joanna C, Wohlfahrt G, Wullschleger Stan D, Woodward FI (2014). The relationship of leaf photosynthetic traits – Vcmax and Jmax – to leaf nitrogen, leaf phosphorus, and specific leaf area: a meta-analysis and modeling study. Ecology and Evolution.

[37] Rai MK, Shende S, Strasser RJ (2008). JIP test for fast fluorescence transients as a rapid and sensitive technique in assessing the effectiveness of arbuscular mycorrhizal fungi in *Zea mays*: analysis of chlorophyll a fluorescence. Plant Biosystems - An Int J Dealing with all Aspects of Plant Biology.

[38] Franken P (2012). The plant strengthening root endophyte *Piriformospora indica*: potential application and the biology behind. Appl Microbiol Biotechnol.

[39] Vadassery J, Tripathi S, Prasad R, Varma A, Oelmuller R (2009). Monodehydroascorbate reductase 2 and dehydroascorbate reductase 5 are crucial for a mutualistic interaction between *Piriformospora indica* and *Arabidopsis*. J Plant Physiol.

[40] Foyer CH, Shigeoka S (2011). Understanding oxidative stress and antioxidant functions to enhance photosynthesis. Plant Physiol.

[41] Mittler R (2002). Oxidative stress, antioxidants and stress tolerance. Trends Plant Sci.

[42] Kumar M, Yadav V, Tuteja N, Johri AK (2009). Antioxidant enzyme activities in maize plants colonized with *Piriformospora indica*. Microbiology.

[43] Hu C, Zhao W, Fan J, Li Z, Yang R, Zhao F, Wang J, Wang S (2015). Protective enzymes and genes related to the JA pathway are involved in the response to root-knot nematodes at high soil temperatures in tomatoes carrying Mi-1. Hortic Environ Biotechnol.

[44] Li X-Y, Gao L, Zhang W-H, Liu J-K, Zhang Y-J, Wang H-Y, Liu D-Q (2015). Characteristic expression of wheat PR5 gene in response to infection by the leaf rust pathogen, *Puccinia triticina*. J Plant Interact.

[45] Waller F, Mukherjee K, Deshmukh SD, Achatz B, Sharma M, Schafer P, Kogel KH (2008). Systemic and local modulation of plant responses by *Piriformospora indica* and related Sebacinales species. J Plant Physiol.

[46] Stein E, Molitor A, Kogel KH, Waller F (2008). Systemic resistance in *Arabidopsis* conferred by the mycorrhizal fungus Piriformospora indica requires jasmonic acid signaling and the cytoplasmic function of NPR1. Plant Cell Physiol.

[47] Vahabi K, Sherameti I, Bakshi M, Mrozinska A, Ludwig A, Reichelt M, Oelmuller R (2015). The interaction of Arabidopsis with *Piriformospora indica* shifts from initial transient stress induced by fungus-released chemical mediators to a mutualistic interaction after physical contact of the two symbionts. BMC Plant Biol.

[48] Hou M, Xu W, Bai H, Liu Y, Li L, Liu L, Liu B, Liu G (2012). Characteristic expression of rice pathogenesis-related proteins in rice leaves during interactions with *Xanthomonas oryzae* pv. Oryzae. Plant Cell Rep.

[49] Bari R, Jones JD (2009). Role of plant hormones in plant defence responses. Plant Mol Biol.

[50] Antico CJ, Colon C, Banks T, Ramonell KM (2012). Insights into the role of jasmonic acid-mediated defenses against necrotrophic and biotrophic fungal pathogens. Front Biol.

[51] Rout E, Nanda S, Joshi RK (2016). Molecular characterization and heterologous expression of a pathogen induced PR5 gene from garlic (*Allium sativum* L.) conferring enhanced resistance to necrotrophic fungi. Eur J Plant Pathol.

[52] Li MSYXF (2014). Jasmonic acid signaling in plants and its biological functions in relation to environment. Shengtai Xuebao/ Acta Ecologica Sinica.

[53] Laudert D, Schaller F, Weiler EW (2000). Transgenic Nicotiana tabacum and *Arabidopsis thaliana* plants overexpressing allene oxide synthase. Planta.

[54] Lorenzo O, Piqueras R, Sanchez-Serrano JJ, Solano R (2003). ETHYLENE RESPONSE FACTOR1 integrates signals from ethylene and jasmonate pathways in plant defense. Plant Cell.

